# Editorial

**DOI:** 10.3897/zookeys.720.22474

**Published:** 2017-12-11

**Authors:** Caroline S. Chaboo, Michael Schmitt

**Affiliations:** 1 Department of Entomology, W-436 Nebraska Hall, University of Nebraska, Lincoln, Nebraska, 68583-0514, USA; 2 Ernst-Moritz-Arndt-Universität, Allgemeine & Systematische Zoologie, Loitzer Str. 26, D-17489 Greifswald, Germany

This special issue assembles a fine collection of authors and recent research that emerged from the 9^th^ International Symposium on the Chrysomelidae, organised within the frame of the 25^th^ International Congress of Entomology, held in September 2016, in Orlando, Florida, USA. This collection of research articles forms the core of volume 7 of Research on Chrysomelidae (RoC), a series that is devoted to all aspects of the biology of leaf and seed beetles. Editor Schmitt has been a co-editor in this series since volume 1 while this is author Chaboo’s first participation as a co-editor.

**Figure:**
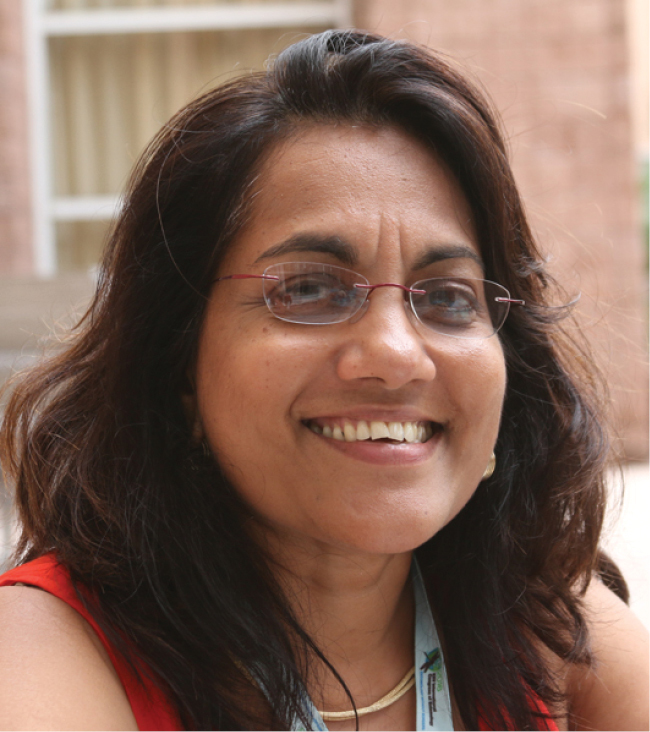


**Figure:**
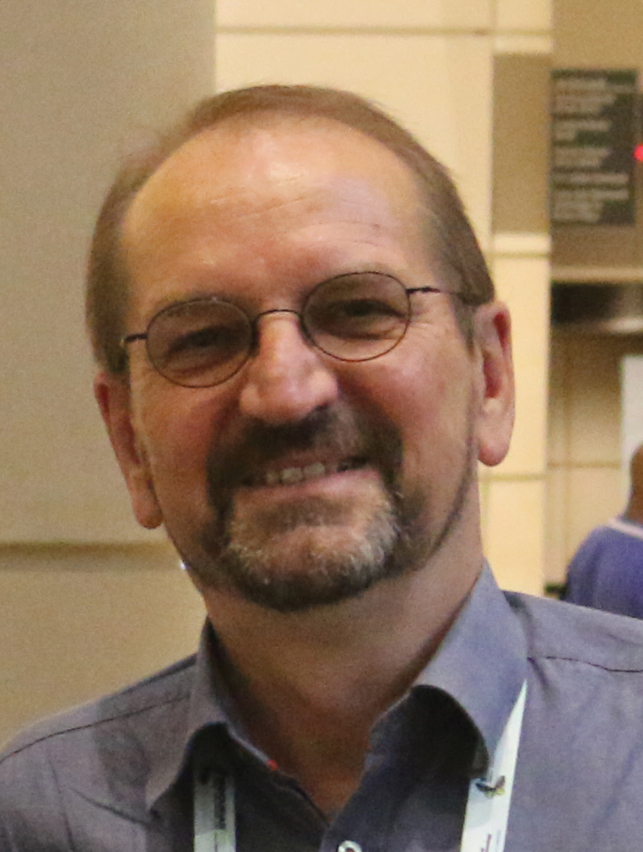


The first volume of the RoC series originated as the brainchild of Pierre Jolivet, starting with volume 1 in 2008 (Editors: Pierre Jolivet, Jorge Santiago-Blay and Michael Schmitt; Brill publishers). The series was intended from conception, and we are happy to be right on target with RoC7 in 2017. Volumes 1 in 2008 and volume 2 in 2009 were presented in independent book formats; since 2011, the chrysomelid community has established a strong working relationship with the ZooKeys publishing team and this has produced volumes 3–6 (2011, 2013, 2015, and 2016). The RoC series mostly contains elaborated versions of research presentations at meeting conferences but also independently submitted papers. Their unconstrained appearance has helped inform and educate on Chrysomelidae systematics and biology.

The issue comprises 9 articles by 23 authors from 9 countries. The majority of articles were presented orally or as posters at the 25^th^ ICE congress. The 9^th^ International Symposium on the Chrysomelidae was the first that also included two contributions on seed beetles (regrettably no manuscripts on seed beetles are included in the present volume). However, we hope this is a change of attitude to include Bruchinae under the umbrella of RoC. Until recently the community of seed beetle workers appeared completely separated from that of leaf beetle workers, reflecting that seed beetles were treated as a separate family, Bruchidae. Since the mode of life of leaf beetles, even of those that are regarded a “pest”, is different from that of seed beetles, the scientists working on the latter had their own agenda. Although the phylogenetic position of the “Bruchidae” within Chrysomelidae was established long ago, treating them as subfamily Bruchinae within the Chrysomelidae became accepted only since 1995. The opposite applies to the Megalopodidae and the Orsodacnidae, both formerly listed as subfamilies within Chrysomelidae. Luckily, leaf beetle workers still include these groups in their field of study, as the paper by Geovanni Rodriguez-Mirón and co-workers shows (pp. 47–64).

Several papers of RoC7 focus on faunistics, biogeography, and biology of leaf beetles in a certain region: Vivan Flinte & co-authors on Rio de Janeiro (pp. 5–22), David Furth on Mexico (pp. 23–46), Yongying Ruan et al. on Chinese flea beetles (pp. 103–120). Some other papers deal with taxonomy: Jesús Gómez-Zurita on Eumolpinae from New Caledonia (pp. 65–75), Rui-E Nie et al. on Galerucinae (pp. 91–102), Michael Schmitt and Gabriele Uhl on Palaearctic *Oulema*-species (pp. 121–130), Thomas Wagner on Afrotropical Galerucinae (pp. 131–137). One contribution deals with functional morphology: Yoko Matsumura and co-authors on traumatic mating in *Pyrrhalta maculicollis* (pp. 77–89).

This broad selection of taxa, topics, and methods demonstrates the attractiveness of leaf beetles as subjects of research in different fields. *Research on Chrysomelidae* provides a forum for diverse and fascinating results on these beetles. We, the editors and the publishers, want to promote further exchange of results and ideas pertaining to all aspects of Chrysomelidae biology among scientists working with different methods in different disciplines, but all on our favourites, the seed and leaf beetles.

Today, chrysomelid researchers and enthusiasts have many ways of sharing their research via the Chrysomela newsletter (established in 1979, over 200 recipients), emails, and social media – Twitter (hashtag #leafbeetles) and a Facebook group ‘Chrysomelidae Forum’ (426 members today). As in 2008, we still believe firmly in the power of meeting face-to-face and in assembling articles in volumes like this one, especially since these provide powerful accelerators for research in a single step.

We look forward to seeing at our upcoming international meetings and in bringing more volumes like the present one into shape.

Caroline S. Chaboo, Michael Schmitt

